# Exercise of Dynamic Stability in the Presence of Perturbations Elicit Fast Improvements of Simulated Fall Recovery and Strength in Older Adults: A Randomized Controlled Trial

**DOI:** 10.3389/fspor.2020.00052

**Published:** 2020-05-27

**Authors:** Sebastian Bohm, Martin Mandla-Liebsch, Falk Mersmann, Adamantios Arampatzis

**Affiliations:** ^1^Department of Training and Movement Sciences, Humboldt-Universität zu Berlin, Berlin, Germany; ^2^Berlin School of Movement Science, Humboldt-Universität zu Berlin, Berlin, Germany

**Keywords:** fall prevention, aging, dynamic stability training, reactive control, unexpected perturbations and disturbances, randomized controlled trial

## Abstract

Age-related impairments of reactive motor responses to postural threats and reduced muscular capacities of the legs are key factors for the higher risk of falling in older people. It has been evidenced that a training of dynamic stability in the presence of perturbations has the potential to improve these deficits. However, the time course of training effects during such interventions is poorly understood. The purpose of this parallel-group study was to investigate the temporal adaptation dynamics of the balance recovery performance and leg strength during a dynamic stability training. Forty-two healthy older adults (65–85 years) were randomly assigned to a training (*n* = 27, analyzed *n* = 18) or control group (*n* = 15, *n* = 14). The training was conducted in a group setting for 6 weeks (3×/week, 45 min). The exercises focused on the mechanism of stability control (i.e., modulation of the base of support and segment counter-rotations around the center of mass) during standing, stepping, and jumping on unstable surfaces with a high balance intensity. Before, after 3 and after 6 weeks, the maximum plantar flexion moment and the knee extension moment were assessed. The recovery performance was evaluated by a simulated forward fall (lean-and-release test) and the margin of stability concept. The margin of stability at release decreased significantly after 3 weeks of training (34%, effect size *g* = 0.79), which indicates fast improvements of balance recovery performance. The margin of stability further decreased after week 6 (53%, *g* = 1.21), yet the difference between weeks 3 and 6 was not significant. Furthermore, the training led to significant increases in the plantar flexion moment after weeks 3 (12%, *g* = 0.72) and 6 (13%, *g* = 0.75) with no significant difference between weeks. For the knee extension moment, a significant increase was found only after week 6 (11%, *g* = 1.07). The control group did not show any significant changes. This study provides evidence that a challenging training of dynamic stability in the presence of perturbations can improve balance recovery performance and leg strength of older adults already after a few weeks. Therefore, short-term training interventions using this paradigm may be an effective strategy for fall prevention in the elderly population, particularly when intervention time is limited.

## Introduction

The increased risk of falling and associated injuries in older adults (Rubenstein, 2006) makes falls a major source of morbidity and mortality (Rubenstein, 2006; Marks, 2011; Alamgir et al., 2012) in a globally senescent population. There is strong evidence that physical exercise interventions can reduce fall risk and rates in older people (Sherrington et al., 2019) and are therefore promoted by international guidelines and national health bodies as a feasible and cost-efficient prevention tool (Moyer, 2012; Kim et al., 2017; Guirguis-Blake et al., 2018; Sherrington et al., 2019).

The age-related decline of muscular capacities in the lower extremities is one intrinsic key factor for the higher risk of falling (Karamanidis et al., 2008; Pijnappels et al., 2008; Graham et al., 2015) and, for that reason, a classical strength training has the potential to improve balance performance (Pijnappels et al., 2008; Arampatzis et al., 2011; Pamukoff et al., 2014). However, the ability to respond to sudden perturbations and postural threats as the cause of a fall event also strongly relies on the successful application of general mechanisms responsible for the dynamic stability control (Bierbaum et al., 2010), i.e., increase in the base of support and counter-rotating segments around the center of mass (CoM) (Hof, 2007). The application of these control mechanisms of the neuromotor system (including perception, signal processing, and motor control) is not actively exercised during classical strength training and likely requires a different intervention approach that focuses on balance recovery performance (Sherrington et al., 2019). In support of this, adaptations of neural control are different after balance compared to strength training (Beck et al., 2007; Gruber et al., 2007; Taube et al., 2007).

Earlier studies of our group showed that when focusing on exercises that promote the application of stability control mechanisms, the ability of older adults to regain balance after unexpected perturbations (which were not explicitly trained) can be improved (Arampatzis et al., 2011; Bierbaum et al., 2013), even beyond the effects of a mixed training approach including resistance training (Bierbaum et al., 2013). However, since in these studies the relative load on the leg muscles was rather low in the stability training group, a strength gain in the lower extremities was not observed. Consequently, we aimed for developing a training that takes advantage of the dynamic stability control exercises but would also increase leg strength in order to target both fall risk factors (i.e., balance recovery performance and muscular capacities) in one intervention and to improve the efficiency of the intervention. For this purpose, we made use of the presence of perturbations evoked by unstable surface conditions that induce continuous variable and partly unpredictable disturbances in combination with the dynamic stability training approach in a subsequent intervention study (Hamed et al., 2018). It has been shown that the presence of perturbations and surface irregularities leads to increased muscle activation, which may effectively stimulate strength gains over time (Munoz-Martel et al., 2019). Furthermore, there is evidence that the presence of fluctuations and disturbances in the neural processing of sensory inputs to motor outputs can improve motor behavior (Faisal et al., 2008; Sejdić and Lipsitz, 2013) and balance performance (Priplata et al., 2003; Aboutorabi et al., 2018; White et al., 2019), and may facilitate motor learning and adaptation (Faisal et al., 2008; Van Hooren et al., 2019). The results of this intervention showed that after 14 weeks of dynamic stability training in the presence of perturbations by unstable surfaces, both strength and balance recovery performance were significantly improved. The increase in balance ability was even greater when compared to a classical resistance training program (Hamed et al., 2018). An association of the exercise-induced changes of the balance recovery performance and changes of the execution time of the recovery step explained the improvement in the stability performance. We concluded that a training, which includes the application of dynamic stability recovery mechanisms in the presence of perturbations, is very effective to improve age-related impairments of the balance recovery performance in fall-like situations.

Yet, little is known about the time course of adaptation of such a training approach. Intervention studies in this field normally use a training period over several months as in our previous study (Hamed et al., 2018). However, in the face of challenging perturbations, either discrete or continuous, the neuromotor system shows acute control adjustments to cope with the postural threat (Bierbaum et al., 2010; Cronin et al., 2013; Graham et al., 2015; Patikas et al., 2016; Santuz et al., 2018; Munoz-Martel et al., 2019), and this ability seems quite unaffected by age (Bohm et al., 2015). It has been suggested that managing such posture-challenging conditions might be a neural mechanism that triggers adaptations of the balance recovery performance (Munoz-Martel et al., 2019). In fact, retention effects have been documented already in days and a few weeks after an initial exposure to specific perturbations (Trimble and Koceja, 2001; Bhatt et al., 2006; Gruber et al., 2007; McCrum et al., 2018). The potential of short-term adaptations is supported by findings of a high temporal plasticity of the motor control system in response to a general balance training (Taube et al., 2008; Taubert et al., 2010; Patel et al., 2019). This suggests that adaptations in the neuromotor control system may improve balance recovery performance and strength capacity already in a short time of systematic exercising (Penzer et al., 2015).

The purpose of the present randomized controlled trial was to investigate the time course of improvements of the balance recovery performance following a simulated forward fall as well as strength capabilities of the plantar flexors and knee extensors in response to a 3- and 6-week intense dynamic stability training in the presence of perturbations. We hypothesized that repeated exposure to continuously variable and partly unpredictable disturbances during the training sessions will lead to improvements in stability recovery performance following a simulated forward fall and muscle strength already after 3 weeks. Furthermore, we hypothesized that the balance recovery improvement can be partly explained by an association of the exercised-induced changes of the stability performance and changes of the execution time of the recovery step.

## Materials and Methods

### Experimental Design

**Figure 1:**
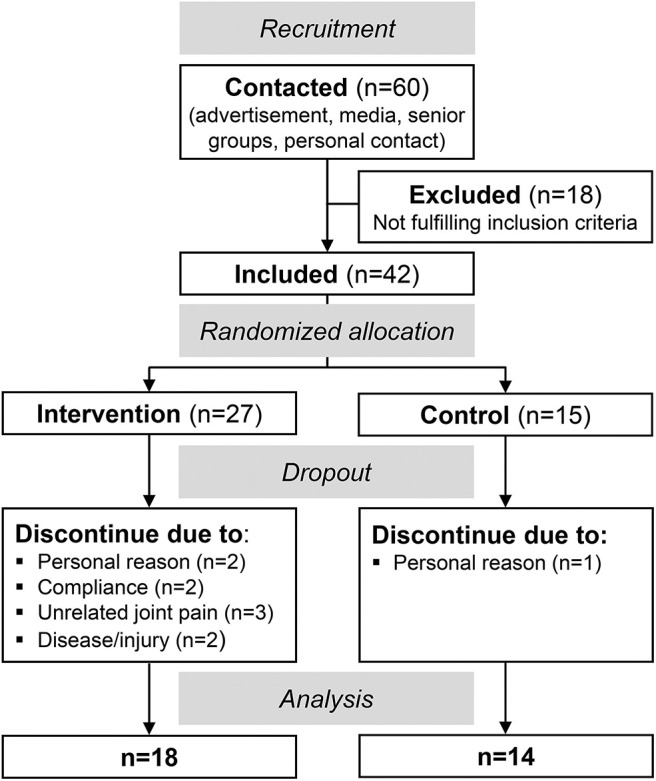
Flow chart illustrating the progress of the intervention trial.

Figure 1 illustrates the progress of the randomized controlled trial with a non-blinded parallel group design. Inclusion criteria for the study were an age between 65 and 85 years and no neural, systemic, and musculoskeletal disorders. Forty-two participants were included and randomly assigned to either a training or control group [simple randomization by a random number generator, 2:1 allocation ratio (intervention/control), Figure 1]. Finally, 32 older adults (>65 years) were analyzed: 18 in the training group (73 ± 6 years, 12 female) and 14 in the control group (73 ± 7 years, 8 female, Figure 1).

The intervention group performed a 6-week training of dynamic stability in the presence of perturbations, based on our earlier study approach (Hamed et al., 2018). The participants of the control group were instructed not to change any of their regular physical activity habits in the intervention period. The primary outcome measures were the balance-recovery performance that was assessed using a simulated forward fall paradigm (lean-and-release test) and the muscle strength of the plantar flexors and knee extensors that was measured on a dynamometer, determined before (week 0), after 3 weeks (week 3), and after 6 weeks (week 6). The ethics committee of the Humboldt-Universität zu Berlin approved the study, and the participants gave written informed consent in accordance with the Declaration of Helsinki. The study followed the CONSORT guidelines (Schulz et al., 2010).

### Exercise Intervention

**Figure 2:**
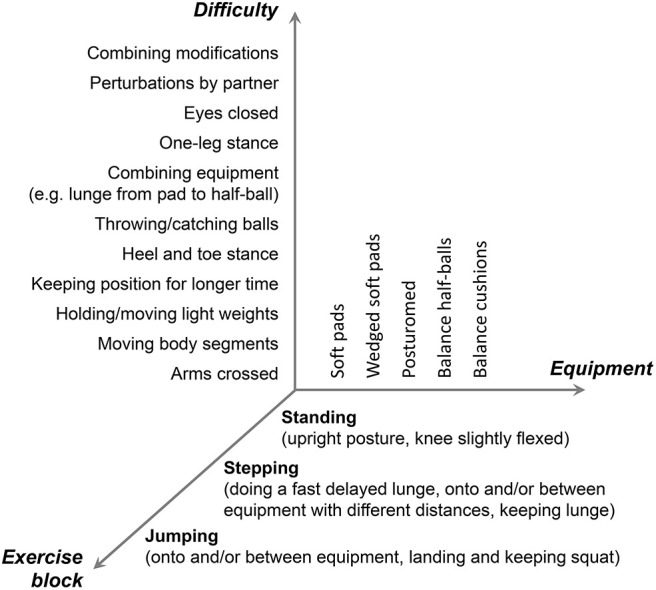
Description of the concept of the training of the dynamic stability in the presence of perturbations. Participants trained each of the exercise blocks under unstable conditions introduced by the different kinds of used equipment. The balance intensity was kept high by modifying the exercises according to the individual performance level to challenge the application of the dynamic control mechanisms (horizontal and vertical axis).

The dynamic stability training was conducted in a supervised group setting taking place in the university's sports gym for 6 weeks, three times a week for 45 min, including a 5-min warm-up and cool-down. The concept of the training is grounded on the exercise of the mechanisms responsible for dynamic stability control (Arampatzis et al., 2011; Bierbaum et al., 2013), i.e., adjusting the base of support and counter-rotations of segments around the CoM (Hof, 2007) in the presence of perturbations (Hamed et al., 2018). Soft, unsteady, uneven, and moveable surfaces were used in order to introduce continuously variable, predictable, and unpredictable perturbations to facilitate balance performance and adaptation. The dynamic stability training program was developed as a three-component approach, where we modified (1) the type of exercise, (2) the used equipment, and (3) the level of difficulty of the exercise (Figure 2). First, the flexible application of the dynamic stability mechanism was challenged during three exercise blocks of standing, stepping, and jumping (Figure 2). Secondly, five different kinds of training equipment [soft pads (Sport-Thieme, balance pad vinyl), wedged soft pads (SoftX, coordination seesaw), posturomed (BIOSWING, Posturomed 202), balance half-balls (Sport-Thieme, balance jumps), and balance cushions (SISSEL, Balancefit)] creating unstable conditions were used to continuously introduce perturbations. Finally, the axis of the level of difficulty of our three-component approach included 11 different modifications of the exercises to increase the balance intensity (e.g., arms crossed, moving body segments, perturbations by partner; Figure 2). The combination of all three components (3 exercise blocks, 5 kinds of equipment, and 11 modifications) resulted in a repertoire of 165 different exercises that have been applied during the intervention. The intensity of the dynamic stability exercise, i.e., postural threat, was progressively adjusted to the individual balance ability level of each participant throughout the training period. The criterion for the instructor to increase the intensity by choosing the next-level exercise (vertical axis in Figure 2) was that the participant could perform a certain exercise without stepping so often off the device or without taking support from a partner (roughly >1 times every 10 s). The exercises were, therefore, not predetermined but adjusted whenever possible. The participants were encouraged by the instructors to focus on keeping the balance during the exercise blocks as good as possible. The instructors guided and supported during the implementation of an exercise modification. The training itself was organized in 5 stations, each with a different kind of training equipment. On the different stations, each exercise block (standing, stepping, and jumping) was trained consecutively. The participants trained pairwise, while the non-exercising partner always stood with arms upright beside the person that trained to give grasping support or to be able to catch the partner when necessary, and mats were placed around the training stations to account for the appropriate safety of the participants. If persons faced any insecurities or felt uncomfortable, the instructor provided further support. After 60 s, the roles of the partners were changed, summing up to about 3 min of training per participant at one station. After 3 and 6 weeks of training, the participants had exercised for about 135 and 270 min, respectively, the mechanism of dynamic stability control in the presence of perturbations at a high level of balance intensity. Because in our last intervention study (Hamed et al., 2018) the training did not lead to significant increases of knee extensor muscle strength, an additional demand to this muscle group was evoked by keeping the knee flexed in the standing position and by maintaining the lunge and squatting position after stepping and landing for a longer time (up to 20 s). Two introduction and familiarization meetings without systematic exercising within 1 week preceded the intervention. We had three consecutive training groups with nine participants who started in each group. Two instructors were present during the training sessions.

### Simulated Forward Fall Paradigm

The participants wore an adjusted upper body harness, which was connected horizontally by a non-elastic rope to an electromagnet mounted on the wall. The magnet was connected in series to a custom-built release system and a force transducer (Megatron 0–5 kN; MEGATRON Elektronik GmbH & Co. KG, Munich, Germany). In this way, the participants were able to lean forward in a straight bodyline, feet hip-width apart and freely hanging arms, while the pulling force expressed as percentage of body weight (BW) was used to control the inclination angle. The initial inclination was set to 8% BW. After receiving a ready cue in the prepared forward lean position, the participants were suddenly released without further warning in an interval of 2–10 s. The participants were instructed to recover a stable lunge with a single step upon the unexpected release. When balance was successfully recovered, the lean angle was increased gradually in 3% BW intervals, until the participants were not able to recover with a single step in three successive attempts. A second rope connected the harness to the ceiling and was adjusted in length, so that the participants would not hit the floor during unsuccessful recovery. The release onset was determined by a 50% reduction in the leaning force signal provided by the force transducer (1,000 Hz). The participants were positioned in such a way that the recovery step by the right leg landed on a force plate (AMTI, BP 400600-2000, 60 cm × 40 cm), and the measured vertical ground reaction force (1,000 Hz) was used for touchdown detection (i.e., increase by ≥5 N).

In order to assess the stability state during release and recovery, we used the concept of the extrapolated CoM proposed by Hof et al. (2005). The extrapolated CoM (X_CoM_) is calculated as:

$$\begin{array}{l} X_{CoM}=\frac{P_{CoM}+V_{CoM}}{\sqrt{\frac{g}{l}}} \end{array}$$

were P_CoM_ is the horizontal (anterior–posterior) component of the projection of the CoM to the ground, V_CoM_ is the horizontal CoM velocity in the same direction, and the term $\sqrt{\frac{g}{l}}$ expresses the eigenfrequency of an inverted pendulum of length l (g is the acceleration of gravity and l is the distance between the CoM and the center of the ankle joint in the sagittal plane). The position of the extrapolated CoM in the anterior–posterior direction was then referred to the anterior boundary (U_max_) of the base of support, expressing the margin of stability (b_x_) (Hof et al., 2005) in the anterior–posterior direction (Karamanidis and Arampatzis, 2007) as:

$$\begin{array}{l} b_{x}=\;U_{\max}-X_{CoM} \end{array}$$

Positive values of the margin of stability, i.e., the extrapolated CoM is within the anterior boundary of the base of support, indicate that the body position is stable, while in the opposite, the stability is lost (Karamanidis et al., 2008). The required kinematic data for the CoM calculation were captured by a Vicon motion capture system (10 cameras at 250 Hz) on the basis of an anatomically referenced set (Bierbaum et al., 2013) of reflective markers (radius 14 mm) that define the respective body segments (foot: calcaneus and second metatarsal bone, shank: lateral femoral epicondyle and lateral malleolus, thigh: greater trochanter and lateral femoral epicondyle, trunk: left and right greater trochanter and C7, upper arm: lateral humeral epicondyle and acromion process, lower arm and hand: midpoint between styloid processes of radius and ulna and lateral humeral epicondyle, head: C7 and a headband with two markers in the front and two in the back). Masses and the locations of the segment CoM were calculated based on the data reported by Dempster et al. (1959), and the body CoM position in the 3D space was calculated according to Winter (1979). The boundaries of the base of support were determined using the vertical projection of the heel marker of the rear foot and the tip of the shoe of the front foot, considering the distance of the metatarsal marker to the anterior boundary of the shoe (measured during preparation).

### Muscle Strength Measurements

The strength of the knee extensors and plantar flexors of the right leg (same as the recovery leg in the simulated forward fall test) was examined during maximum voluntary isometric contractions (MVC) on a Biodex dynamometer (Biodex Medical, Syst.3, Shirley, NY, USA). For the knee extensions, the participants were seated on the dynamometer chair with a trunk angle flexion of 85° (trunk in line to the thighs = 0°) and for the plantar flexions with an angle of 70° and with fully extended knee, while the arms being crossed on the chest. Following a standardized warm-up, five MVCs in different joint angles were performed for the knee (between 50 and 75°) and the ankle joint (between 8 and 25° dorsiflexion), respectively. A 3-min rest was given between trials, and the highest value was used for further analysis. The resultant ankle and knee joint moments were calculated using an established inverse dynamics approach in order to account for axis misalignment between the dynamometer and the joint as well as gravitational moments (Arampatzis et al., 2004, 2005). For this purpose, anatomically referenced reflective markers (greater trochanter, medial and lateral femoral epicondyles and malleoli, second metatarsal bone, and calcaneus bone) were captured using a Vicon motion analysis system (Version 1.8, Vicon Motion Systems, Oxford, UK) integrating seven cameras at 250 Hz.

### Statistics

A statistical power analysis was performed *a priori* to calculate the required sample size by means of the software G^*^Power (version 3.1.9.6, Germany). For this purpose, we used the effect size of the margin of stability at release from our previous dynamic stability training intervention study (Hamed et al., 2018). Since the training frequency per week was higher while the intervention duration of the current study was shorter, we assumed a reduced effect (by ~20%). The power analysis was conducted for the *post-hoc* time point comparison for the intervention group, considering a Bonferroni correction of the *p*-values [α = 0.0167 (adjusted), power = 0.8, effect size: 0.86, two-tailed], and revealed a sample size of *n* = 17. We included 27 participants for the intervention group to consider possible dropouts.

An analysis of variance for repeated measures on a linear mixed model was calculated for the strength (normalized to body weight) and stability parameters with the time point as the within-subjects factor (week 0 vs. week 3 vs. week 6) and group as a between-subjects factor (intervention vs. control). In case of time effects or time by group interactions, a Benjamini–Hochberg corrected *post-hoc* analysis was conducted separately for each group (adjusted *p*-values will be reported). Baseline (week 0) anthropometric, strength, and stability parameters were compared between the intervention and control group using the same linear mixed model. The relationship between the changes in the margin of stability at release and the changes in the rate of increase in the base of support from release to touchdown was analyzed by means of a Pearson correlation coefficient. The level of significance was set to α = 0.05, and statistical analyses were conducted using R v3.4.1 (R Found. for Stat. Comp.). Effect sizes (Hedges' g) were calculated to assess the strength of the intervention effects, where 0.2 ≤ *g* < 0.5 indicates small, 0.5 ≤ *g* < 0.8 indicates medium, and *g* ≥ 0.8 indicates a large effect size (Cohen, 1988). Note that due to technical or personal issues, some datasets from the three different measurement time points of the intervention and control groups of the two strength tests (25 from 192) and stability test (15 from 96) were missing; however, a strength of linear mixed models is that they can handle missing data and, thus, the respective participants could be included.

## Results

Sixty older adults (>65 years) were contacted and finally 42 meet the inclusion criteria and agreed to participate (Figure 1). Twenty-seven of those were randomly assigned to the intervention group and 15 to the control group. Ten participants discontinued the intervention (nine dropouts in the intervention and one in the control group) and, thus, 18 participants of the intervention group and 14 of the control group were included in the final analysis (Figure 1). There were no baseline differences of the margin of stability at release and the maximal ankle and knee joint moment between those who completed the training and those who dropped out (*p* > 0.05). The intervention and control group did not show any significant differences with respect to age (intervention: 73 ± 6 years, control: 73 ± 7 years, *p* = 0.903), height (164 ± 11 cm, 169 ± 10 cm, *p* = 0.200), and body mass (70 ± 15 kg, 69 ± 10 kg, *p* = 0.970). A baseline (week 0) group difference was found for the margin of stability at release (*p* = 0.018) and for the rate of increase in the base of support (*p* = 0.007, Table 1).

**Table 1:** Outcome parameters before (week 0), within (week 3), and after (week 6) the training period for the intervention and control groups.

**Parameter**	**Intervention group**			**Control group**		
	**Week 0**	**Week 3** **(g: w0–3)**	**Week 6** **(g: w0–6, w3–6)**	**Week 0**	**Week 3** **(g: w0–3)**	**Week 6** **(g: w0–6, w3–6)**
MoS RS (cm)^*^	−8.19 ± 6.56	−10.97 ± 6.40 (−0.79)^#^	−12.55 ± 6.24 (−1.21, −0.44)^#^	−14.09 ± 6.55'	−14.64 ± 6.55 (−0.16)	−15.97 ± 6.17 (−0.51, −0.36)
MoS TD (cm)	11.44 ± 5.94	10.17 ± 5.88 (−0.22)	8.64 ± 5.81 (−0.49, −0.27)	7.24 ± 5.95	8.21 ± 5.95 (0.18)	7.29 ± 5.75 (0.01, −0.16)
BoS TD (cm)	95.3 ± 14.6	101.1 ± 14.3 (0.62)	100.0 ± 13.9 (0.50, −0.11)	103.9 ± 14.6	103.5 ± 14.6 (−0.05)	106.9 ± 13.8 (0.30, 0.35)
Duration RS-TD (ms)^~^	524.7 ± 63.5	552.0 ± 63.6 (0.31)	498.0 ± 63.5 (−0.30, −0.60)	479.7 ± 63.6	472.9 ± 63.6 (−0.08)	475.6 ± 63.4 (−0.05, 0.02)
Rate of BoS (cm/s)^*^^~^	182.2 ± 32.9	189.9 ± 32.2 (0.34)	200.0 ± 31.5 (0.77, 0.44)^#^	216.8 ± 32.9'	218.9 ± 32.9 (0.10)	224.5 ± 31.2 (0.33, 0.24)
Moment ankle (Nm/kg)^*^	1.57 ± 0.44	1.76 ± 0.46 (0.72)^#^	1.78 ± 0.43 (0.75, 0.08)^#^	1.65 ± 0.47	1.72 ± 0.47 (0.30)	1.78 ± 0.43 (0.50, 0.22)
Moment knee (Nm/kg)^*^	2.00 ± 0.45	2.10 ± 0.44 (0.60)	2.21 ± 0.40 (1.07, 0.54)^#^	2.32 ± 0.43	2.38 ± 0.45 (0.39)	2.39 ± 0.42 (0.38, 0.01)

*Mean ± SD of the margin of stability (MoS) at release (RS), margin of stability and base of support (BoS) at touchdown (TD), duration from release until touchdown, rate of increase in the base of support (Rate of BoS), maximum voluntary isometric ankle plantar flexion moment and knee extension moment (normalized to body weight); g, Hedges' g effect size (note that negative values for the MoS RS and Duration RS-TD indicate a positive performance effect)*.

* *Statistically significant time effect (p < 0.05)*.

# *Statistically significant difference (post-hoc analysis) to week 0 (p < 0.05)*.

~ *Statistically significant group effect (p < 0.05)*.

‘ *Statistically significant difference (post-hoc analysis) to the intervention group (p < 0.05)*.

**Figure 3:**
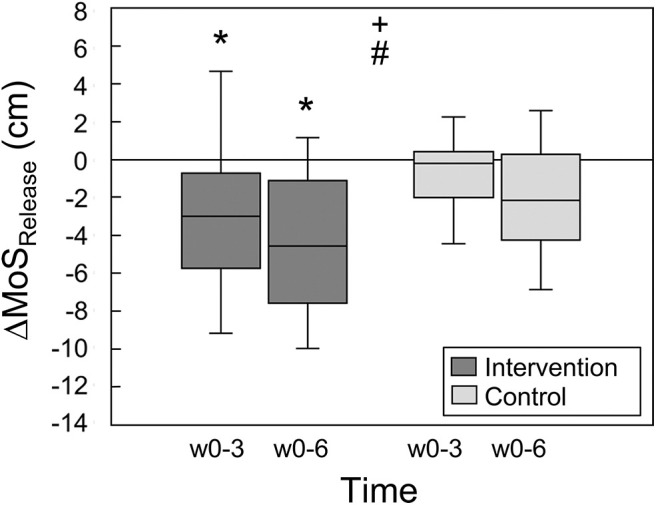
Changes of the margin of stability at release (ΔMoS_Release_) during the simulated forward falls between weeks 0 and 3 as well as weeks 0 and 6 for the intervention (*n* = 18) and control groups (*n* = 14), respectively. Statistically significant main effect of + time and # group (*p* < 0.05). *Statistically significant difference (*post-hoc* analysis) to baseline, i.e., week 0 (*p* < 0.05).

The margin of stability at release showed a significant main effect of time (*p* < 0.001) and no significant time by group interaction effect (*p* = 0.098). The intervention group showed a significant decrease in the margin of stability at release from week 0 to week 3 (*p* = 0.013) and week 0 to week 6 (*p* < 0.001), but no significant change from week 3 to week 6 (*p* = 0.258, Table 1). This indicates fast improvements in balance recovery performance after a short time of training. The control group showed no significant differences between the time points (*p* > 0.05). Figure 3 illustrates the changes between weeks 0 and 3 as well as weeks 3 and 6 in the margin of stability at release, which were significantly greater in the intervention group compared to the control group (*p* = 0.044).

**Figure 4:**
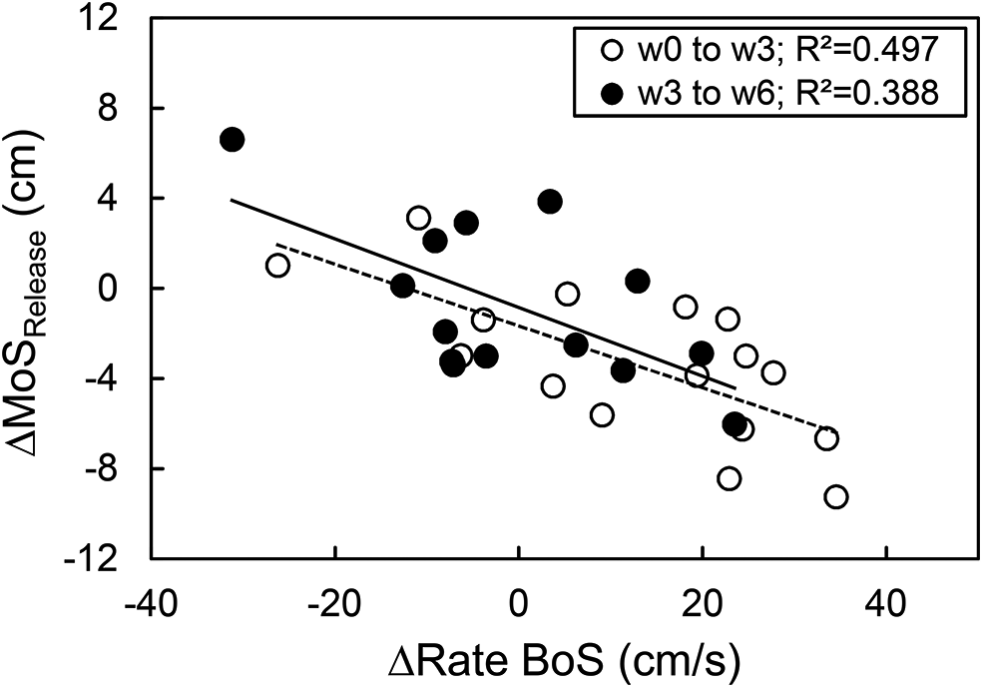
Relationship between the exercise-induced change of the margin of stability at release (ΔMoS_Release_) and the rate of the base of support increase (ΔRate BoS) from release to touchdown during the simulated forward fall for the time course of week 0 to week 3 (*n* = 16) and week 3 to week 6 (*n* = 14).

The base of support of the recovery step in the anterior direction showed no significant time by group interaction effect (*p* = 0.158) and no significant time effect (*p* = 0.056), hence no significant differences were found for both groups between time points (*p* > 0.05, Table 1). A significant time effect (*p* = 0.008) but no time by group interaction effect (*p* = 0.471) was observed for the rate of increase in the base of support from release to touchdown. For the intervention group, a significant increase in the rate from weeks 0 to 6 was found (*p* = 0.034, Table 1). No further significant differences were observed for the other time points and the control group (*p* > 0.05). There was a significant correlation between the changes in the margin of stability at release and the changes in the rate of increase in the base of support from release to touchdown between weeks 0 and 3 (*r* = −0.705, *p* = 0.002), weeks 0 and 6 (*r* = −0.733, *p* = 0.001), and weeks 3 and 6 (*r* = −0.623, *p* = 0.017, Figure 4).

The ankle and knee joint moments showed a significant main effect of time (*p* = 0.002, *p* < 0.001) but no significant time by group interaction (*p* = 0.499, *p* = 0.124), respectively. The ankle joint moment of the intervention group revealed a significant increase from week 0 to week 3 (*p* = 0.041) and week 0 to week 6 (*p* = 0.041), but no significant change from week 3 to week 6 (*p* = 0.947, Table 1). The knee joint moment was not significantly different between weeks 0 and 3 (*p* = 0.131) and weeks 3 and 6 (*p* = 0.156) but was significantly different between week 0 and week 6 (*p* < 0.001, Table 1). No significant time point differences were observed for the ankle and knee joint moment of the control group (*p* > 0.05, Table 1).

## Discussion

In the present study, we investigated the initial time course of adaptive responses to a challenging training of the dynamic stability in the presence of perturbations, considering the effects on balance recovery performance and lower limb strength capacities of older adults. In agreement with our hypotheses, we found improvements in recovery performance and strength already after 3 weeks of intervention, suggesting fast and effective improvements of critical fall risk factors.

The results showed that a few sessions (nine training sessions) of a stability training in the presence of perturbations, which included the application of mechanisms responsible for dynamic stability control under unstable conditions, were sufficient to improve the reactive stepping behavior during a simulated forward fall test. The recovery performance, defined as the lowest margin of stability at release (or most unstable body position) that could be recovered with a single step, improved by 34% (*g* = −0.79) after week 3 (nine training sessions) and by 53% (*g* = −1.21) after week 6 (18 training sessions) in the intervention group. Reactive stepping performance following a forward loss of balance was shown to be a predictor of the risk of falling (Carty et al., 2015; Okubo et al., 2017). Therefore, the finding of fast improvements in stability performance promotes the application of the current short-term dynamic stability training approach for the prevention of falling in the older population. Furthermore, the training can be applied in a group setting under the supervision of one or two instructors and with cheap and conventional equipment, making this approach very feasible and attractive for a clinical and broader setting.

The improved balance recovery performance in the intervention group was associated with a higher rate of increase in the base of support from release to touchdown, i.e., faster recovery step. Although the gain of the rate of increase in the base of support was only significant after week 6, changes in the rate of increase in the base of support were inversely correlated with changes in the margin of stability at release at all time points. The base of support of the recovery step did not show any significant changes after weeks 3 and 6. This confirms our previous findings underlining the importance of the ability to execute recovery steps in a short time for balance recovery (Karamanidis et al., 2008; Hamed et al., 2018). However, the delayed significant increase in the rate of increase in the base of support (after week 6) compared to the earlier changes of the margin of stability at release (after week 3) may indicate that exercise-induced alterations of other mechanisms (e.g., counter-rotation of segments) also contributed to the improvements of the balance recovery performance, particularly in the initial phase of the intervention (i.e., first weeks).

The fast improvements in the simulated forward fall test following the current training of the dynamic stability in the presence of perturbations, in which this testing task was not explicitly exercised, may rely on the changes of the control by the neuromotor system when coping with postural challenges. Recently, we found that in the presence of perturbations, the basic activation patterns of muscle groups in both balance and locomotion tasks become fuzzier (Santuz et al., 2018, 2020; Munoz-Martel et al., 2019), less unstable, and less complex (Santuz et al., 2020), indicating increased control robustness (i.e., ability to cope with errors; Santuz et al., 2018) in challenging settings. The perturbation-induced modification and modulation of activation patterns might be a neural mechanism that improves dynamic stability performance when repetitively applied such as during the present training of dynamic stability (Munoz-Martel et al., 2019). Furthermore, the presence of continuous disturbances in the neural processing of sensory inputs to motor outputs can improve balance performance (Priplata et al., 2003; Aboutorabi et al., 2018; White et al., 2019), and may stimulate motor adaptation (Faisal et al., 2008; Van Hooren et al., 2019) resulting in the enhanced balance recovery ability.

After 3 and 6 weeks of intervention, we observed significant increases in plantar flexor (*g* = 0.72, *g* = 0.75) and knee extensor muscular (*g* = 0.60, *g* = 1.07) capacities, respectively. Challenging postural settings change the activation patterns of the lower limb muscles with higher and longer activations to generate compensatory joint moments (Cheung et al., 2009; Cronin et al., 2013; Voloshina et al., 2013; Voloshina and Ferris, 2015; Nazifi et al., 2017; Santuz et al., 2018; Munoz-Martel et al., 2019). During a single training session, the participants were exposed to about 15 min of continuous perturbations at a high balance intensity with body positions that put a great demand on the control and the muscular system. This summed up to about 135 min after 3 weeks and 270 min after 6 weeks. Neural plasticity can take place in a very short time on the spinal, supraspinal, up to the cortical level in response to short-term balance (Taube et al., 2008; Taubert et al., 2010; Patel et al., 2019) and strength training (Carroll et al., 2002, 2011), or a combination of both (Penzer et al., 2015). Thus, we can argue that the presence of perturbations during the training likely triggered changes in the neural drive to the muscle over time and consequently caused the observed strength gains.

The increase in muscle strength of the plantar flexors became significant already after 3 weeks of training (week 3: 12%, week 6: 13%), while for the knee extensors, a significant increase was first observed after week 6 (11%). The earlier gains in the plantar flexors might be due to the fact that the majority of the exercise blocks were performed on soft surfaces (soft pads, balance half-balls, balance cushions) in which predominantly the plantar flexors are involved to maintain anterior–posterior balance. Furthermore, it has been suggested that the plantar flexors are sensitive for surface perturbations due to their morphological design (longer tendons and shorter fascicles) and direct interaction with the ground (Biewener and Daley, 2007; Daley et al., 2007), leading to higher activation levels and presumably faster adaptive responses (Hamed et al., 2018).

In our previous study with the same approach, we were not able to provoke significant increases in the knee extensor strength (*d* = 0.41) (Hamed et al., 2018). Given the importance of the knee extensors for balance recovery (Karamanidis and Arampatzis, 2007; Karamanidis et al., 2008), we modified the exercises for the present intervention to involve this muscle group more in the stabilizing task and to increase the loading demand. This was achieved by introducing more flexed knee angles during the three exercise blocks and by keeping a more lunged/squatted position for several moments after the step and jump on the unstable training utensils. The significant increase in the knee extensor strength after week 6 indicates that the simple modification of the exercises was effective and led to strength gains comparable to the plantar flexors after 6 weeks of intervention, i.e., plantar flexors 13% (*g* = 0.75) and knee extensors 11% (*g* = 1.07).

The balance recovery performance and plantar flexor strength were significantly increased after week 3 with no further significant increase after week 6. This indicates that in the time course of the current training of the dynamic stability in the presence of perturbations, adaptive response rates seem to be higher in the first weeks when compared to the following time period. In our previous intervention study with a similar training approach, the participants exercised twice a week for 14 weeks (Hamed et al., 2018). Despite the lower frequency, the longer intervention period provoked notably greater improvements, i.e., 80% for the forward fall test and 20% for the strength measurement compared to 53% and 13% after week 6 in the present study. Thus, longer training periods seem to be beneficial to promote adaptive effects in older adults.

Although the participants were randomly assigned, we found baseline differences between the intervention and control groups for the margin of stability at release and the rate of increase in the base of support. This was mainly due to two participants of the control group, which performed exceptionally well in the balance recovery test. Both reported to be physically active, which may explain the performance above average. When excluding these two datasets from the analysis, the differences diminished. Since no further group comparisons were made, the participants were included to increase the statistical power. In the current study, we faced a comparably high dropout rate (33%). However, as can be seen in Figure 1, the reasons for the dropout were not related to the intervention itself, i.e., unrelated joint pain (*n* = 3), diseases (*n* = 2), and personal reasons (*n* = 2), while two participants were withdrawn because of limited training compliance. For the last two reasons, it should be noted that this was a voluntary participation with no compensation, but the time efforts for the measurements (three times with about 3 h of testing) in addition to the time spent for the training were quite high. Therefore, we can assume that the training itself is indeed feasible and not too difficult or overchallenging. Furthermore, we included only healthy older adults in the intervention and, therefore, any translation of the findings to other populations, e.g., frailty or pathologies, warrants further investigation. No clinical trial registration had been performed.

In conclusion, the current study provides evidence that a challenging stability training, which focuses on the application of mechanisms responsible for dynamic stability control in the presence of perturbations, can improve balance recovery performance and leg strength already after a short time period, i.e., 3 weeks. Therefore, short-term training interventions using this exercise paradigm may be an effective strategy for fall prevention, particularly when intervention time is limited.

## Data Availability Statement

The datasets generated for this study are available on request to the corresponding author.

## Ethics Statement

The studies involving human participants were reviewed and approved by Ethics committee of the Humboldt-Universität zu Berlin. The patients/participants provided their written informed consent to participate in this study.

## Author Contributions

SB and AA designed the research and drafted the manuscript. SB and MM-L performed the research and analyzed the data. FM and MM-L made important intellectual contributions during revision.

## Conflict of Interest

The authors declare that the research was conducted in the absence of any commercial or financial relationships that could be construed as a potential conflict of interest.
